# *Veratrilla baillonii* Franch Ameliorates Diabetic Liver Injury by Alleviating Insulin Resistance in Rats

**DOI:** 10.3389/fphar.2021.775563

**Published:** 2021-11-26

**Authors:** Zhi-Hao Zhang, Juan Li, Jun Li, Zhaowu Ma, Xian-Ju Huang

**Affiliations:** ^1^ College of Pharmacy, South-Central University for Nationalities, Wuhan, China; ^2^ Key Laboratory of Environmental Health, Ministry of Education, Department of Toxicology, School of Public Health, Tongji Medical College, Huazhong University of Science and Technology, Wuhan, China; ^3^ School of Basic Medicine, Health Science Center, Yangtze University, Jingzhou, China

**Keywords:** *Veratrilla baillonii* franch, diabetes mellitus, insulin resistance, diabetic liver injury, transcriptome

## Abstract

Type 2 diabetes mellitus (T2DM) is a complex and polygenic disorder with diverse complications. *Veratrilla baillonii* Franch (*V. baillonii*) has been applied in the intervention and treatment a diverse range of diseases, including diabetes. In this study, we revealed that water extracts of *V. baillonii* (WVBF) can ameliorate liver injury and insulin resistance in T2DM rat model. To elucidate the anti-diabetic mechanisms of WVBF, we performed liver transcriptome analysis that displayed WVBF treatment significantly suppressed many gene expressions involved in insulin resistance. Furthermore, functional experiments showed that WVBF treatment reduced the pathological damages of liver and pancreas, which may be regulated by Foxo1, Sirt1, G6pc, c-Met, Irs1, Akt1, Pik3r1. These results indicated that WVBF improves diabetic liver injury and insulin resistance in diabetic rats. Therefore, this study demonstrated WVBF could be used as a promising therapeutic agent for intervention and treatment of diabetes.

## Introduction

T2DM (type 2 diabetes mellitus) is a metabolic disorders-induced chronic illness, which cause abnormal glucose, lipid and protein metabolism (Pandey et al., 2015). As evidenced by the literature, the T2DM is characterized by progressive *ß* cell failure and insulin resistance (Cinti et al., 2016). In type 2 diabetes, a decline in insulin action termed insulin resistance, followed by the inability of *ß*-cells to secrete enough insulin to compensate for the insulin resistance, is considered as the initial event leading to the developmental process of T2DM (King et al., 2016). An accumulating body of evidence indicated that insulin resistance can strengthen the pathological progression of multi-organ impairments, including kidney and liver in patients (Lontchi-Yimagou et al., 2013). Inflammation, together with disturbance of systemic and hepatic fat metabolism, is mainly regarded as causative factor in diabetic liver injury (Boden et al., 2005). Hyperglycemia increases the production of free radicals (Gutteridge and Halliwell, 1992) and induces oxidative stress that trigger liver injuries with carbohydrate metabolism disorder (Loguercio and Federico, 2003; Vitaglione et al., 2004). Accumulating studies uncovered that the major molecules involved in metabolic disorder associated with T2DM include insulin signaling and related molecules (Tian et al., 2013).

The gentian family, consisting of 99 genera with a total of about 1736 species, are plants with medicinal uses to treat many diseases involved in metabolic disorders (Tomiczak et al., 2019). *Swertia corymbosa*, a plant in the family Gentianaceae and used in traditional medicine to treat diabetes in Indian (Mahendran et al., 2014). The ethanol extract of *Swertia kouitchensis* has been shown to inhibit α-amylase and α-glucosidase, which was effective to reduce blood glucose levels in STZ-induced diabetic mice (Wan et al., 2013). Extract of the milled aerial parts of the dried plant of *Centaurium erythraea*, which at 250 mg/kg body weight has been shown to reduce hyperglycemia and hyperlipidemia in STZ-induced diabetic rats (Stefkov et al., 2014). Currently, 417 species of genus Gentian are identified in China, which are distributed in most of provinces and regions (Liu et al., 2016). Gentiopicroside, sweroside, and swertiamarin are the characteristic components of the medicinal plants of Gentianaceae used in the practice of traditional Chinese Medicine. Our previous study identified that WVBF are rich in sweroside, swertiamarin, gentiopicroside (GP) (Ma et al., 2018), which can exert anti-hepatotoxic and hepatoprotective effects (Dai et al., 2018a; Li et al., 2019). Among many Chinese herbal extracts with hepatoprotective activity, the extracts of gentian plants endow uniquely advantages, including hepatoprotection and rescue of liver injury (Zhao et al., 2012). Our previous work confirmed that gentiopicroside and sweroside, the responsible bioactive compounds, activate insulin signal transduction cascade involved in hepatic metabolism (Huang et al., 2016). The content of sweroside and gentiopicroside from WVBF was 0.36 and 3.75%, respectively (Ma et al., 2018). *Veratrilla baillonii* Franch (*V. baillonii*), a member of Gentianaceae family, is characterized by anti-toxic and liver protection properties (Li et al., 2019), and used in the intervention of drug-induced hepatitis and hepatitis-induced jaundice (Olennikov et al., 2015; Zhang et al., 2015). The water extract of *V. baillonii* (WVBF) has significant protective effects on oxidative stress-induced liver injury, diabetic liver injury, and drug induced liver toxicity (Lian et al., 2010; Huang et al., 2016; Huang et al., 2020).

Our previous work has identified that WVBF could attenuate hepatic toxicity induced by Aconitum brachypodum Diels (Ranunculaceae) *in vivo* and *in vitro* (Ge et al., 2016; Yu et al., 2016; Dai et al., 2018b; Cheng et al., 2019; Huang et al., 2019; Li et al., 2019). Further study revealed that the anti-diabetic and hepatic protection activities of WVBF is mediated by IRS-1/PI3K/AKT signal pathway in T2DM db/db mice. (He et al., 2020a). However, the whole interaction network of WVBF on diabetic liver injury remains unclear and need further discussed. The present study was thus conducted to validated the protective effect of WVBF against liver injury in diabetic rats induced by a high-fat diet and streptozocin (HFD-STZ) injection. The clinic features as well as blood glucose and insulin resistance were observed to evaluate the efficiency. The pathology of liver and pancreas were evaluated by Hematoxylin and Eosin (H&E) Staining. This present study aimed to explore the underlying mechanisms of WVBF in diabetes, we analyzed the transcriptome profiling of the T2DM rats liver in the presence or absence of WVBF. The key target genes of dysregulated pathways related to T2DM were detected by qRT-PCR method. These results could give a systematic explanation of the protective effect of WVBF on T2DM induced liver damage.

## Materials and Methods

### Materials

TRIzol was obtained from Tiangen Biochemical Technology Co., Ltd. (Beijing, China); Streptozotocin was obtained from Saiguo Biotechnology Co., Ltd. (Guangzhou, China); Blood glucose test paper and blood glucometer purchased from Sinocare Biosensor co. (Changsha, China); Rat Insulin ELISA kit was obtained from Biyuntian Biological Technology Co., Ltd.

### Animals

Sprague-Dawley (SD) rats, male, 8 weeks old (Yu et al., 2019), weighing 160–180 g, purchased from the Experimental Animal Center of Huazhong Agricultural University, license number: SCXK- (Liao) 2015-0001. Animal welfare and experimental procedures were performed as described previously in our lab (He et al., 2020a). Rats were housed and maintained under a controlled environment (22 ± 2°C, 60 ± 10% humidity) with a 12:12 h light: dark cycle. The experiment started 1 week after the rats were acclimatized. 60 rats were randomly divided into 8 normal control group (NC) and fed with normal diet. Except for the NC group, the remaining rats were fed high-fat diet (HFD, 88% of normal diet, plus 2% of cholesterol and 10% of lard) for 4 weeks for T2DM modeling. After 4 weeks of feeding (with body weight around 350 g and fasting blood glucose (FBG) around 5 mmol/L), rats were intraperitoneally injected with STZ (40 mg/kg) (Li J.-c. et al., 2018) to induce the T2DM model, and then treated with normal diet for 6 weeks. Animals whose fasting blood sugar level was higher than 11.1 mmol/L seven days after STZ injection were successfully molded. 45 of the 52 rats injected with STZ were successfully established the model. Diabetes rats were divided into the following four groups: Group one was reserved as mellitus control (MC) group (*n* = 8), group two rats were administrated intragastrically with Metformin Hydrochloride 150 mg/kg (Abu Bakar Sajak et al., 2017) positive (PC) group (*n* = 8) once a day for 6 weeks; group three and four rats were administrated intragastrically with WVBF 12.5 mg/kg (*n* = 8) and 25 mg/kg (Huang et al., 2020) (*n* = 8) once a day for 6 weeks, respectively. The NC group and the MC group were orally given 0.9% normal saline according to their body weight. The PC group, WVBF 12.5 mg/kg group, and WVBF 25 mg/kg group were weighed according to their body weight, dissolved with 0.9% normal saline and administered orally.

### Total RNA Extraction and RNA-Seq Analysis

Three liver samples were collected from NC, MC, WVBF 12.5 mg/kg and 25 mg/kg each group. After overnight fasting for 12 h, liver tissues were collected. rinsed with diethyl pyrocarbonate (DEPC) water prepared with phosphate buffer saline (PBS), frozen in liquid nitrogen for 30 s, transferred to enzyme-free EP tube and frozen at -80°C. Total RNA was isolated using TRIzol reagent (Ambion, United States) according to the manufacturer’s protocol. The concentration, quality and integrity were determined using a Nanodrop spectrophotometer (BioDrop Technologies, UK) and an Agilent bioanalyzer 2100 (Agilent, United States).

The sequenced reads obtained by sequencing contain splice sequences and low-quality reads. In order to ensure the quality of information analysis, raw reads need to be filtered to obtain clean reads. Bioacme adopts Fastp version 0.20 for quality filtering. 1) Remove the connector sequence (adapter); 2) Remove reads with low quality (mass value less than 15) alkali base exceeding 40% of the length proportion of the read; 3) Remove reads with base number of “n” base exceeding 5; 4) Start from the 5 “end of reads (i.e. the beginning of reads), filter the sliding window quality, and cut off the sliding window (sliding window size 4) whose average base quality value is lower than the threshold (20); 5) Remove the filtered reads whose length is less than 36. FeatureCounts version 1.5.0 was used to obtain the fragments per kilobase of exon per million fragments mapped (FPKM) of all annotated genes. DEGs were identified using DESeq2 with the default parameters at *p* < 0.05 for the four groups. A total of 12 cDNA libraries were generated by the TruSeq RNA sample preparation kit (Illumina, United States). Briefly, mRNA was purified from total RNA using poly-T oligo-attached magnetic beads. The established sequencing library was sequenced with Illumina Hiseq novaseq 6000 by whbioacme. Differential expression analysis of RNA-seq data was performed as described in previous study (Feng et al., 2012; Liao et al., 2014; Love et al., 2014). Venn diagram of differentially expressed genes (DEGs) in the different groups: MC versus WVBF 25 mg/kg, MC versus WVBF 12.5 mg/kg, WVBF 25 mg/kg versus WVBF 12.5 mg/kg, and MC versus NC.

### Measurements of FBG

FBG was monitored at 0, 1, 2, 3, 4, 5 and 6 weeks after-treatments of diabetic and normal rats. Blood was collected from the tail of the animals after 12–16 h overnight fasting. The tail tip was sanitized with alcohol and pricked and a drop of blood was used for blood glucose measurement using a glucometer (Sinocare Biosensor Co., Ltd., Changsha, HN, China). FBG was presented by a broken line graph of weekly blood glucose dynamic changes.

### Oral Glucose Tolerance Test

OGTT was performed in rats as described previously in our lab after 6 weeks of WVBF treatment (He et al., 2020a). Briefly, 50% glucose solution were administrated to overnight-fasted rats by oral gavage at a dose of 2 g/kg. The blood glucose concentrations was measured using glucometer at 0, 30, 60, 90, and 120 min respectively after the glucose gavage.

### Homeostatic Model Assessment of Insulin Resistance (HOMA-IR) and HOMA- *ß* Cell Function Index

Insulin resistance index, HOMA-IR, was assessed using fasting glucose ( mmol/L) × fasting insulin ( mμ/L)/22.5. HOMA-β cell function index, HOMA-β, was assessed using 20×fasting insulin ( mμ/L)/fasting glucose (mmol/L)-3.5.

### Histological Analysis

Liver and pancreas tissues were removed, fixed with 10% neutral buffered formalin over-night, embedded in paraffin, and sectioned at 5 μm. Next, H&E staining was were performed as previously described in detail in our lab (He et al., 2020a).

### Total RNA Extraction and Real-Time qPCR Analysis

Total RNA was extracted from rat liver tissues with TRIZOL reagent (Tiangen, China). The concentration and purity of different RNA samples were evaluated using a spectrophotometer (Thermo Fisher Scientific, Waltham, MA, United States).The cDNA was synthesized by reverse transcriptase (Tiangen, China). Real-time qPCR was performed using the Biosystems 7500 Fast Real-Time PCR System (Thermo Fisher Scientific (China) Co., Ltd.). The primers of real-time qPCR were used to amplify transcripts for c-Met, Irs1, Akt1, Pik3r1, Foxo1, Sirt1 and G6pc (Table 1). *ß*-actin served as the endogenous housekeeping gene to normalize the expression of the RNA sample levels. Relative gene expression levels were calculated using the 2^−ΔΔCT^ method.

**TABLE 1:** The primers used for RT-PCR in this study.

Gene name	Primer sequence
*β-actin*	Forward 5′-TGA​CAG​GAT​GCA​GAA​GGA​GAT​TAC - 3'
	Reverse 5′-GAG​CCA​CCA​ATC​CAC​ACA​GA - 3′
*c-Met*	Forward 5′-CTG​ACG​AGT​GGA​GAC​TCT​GAT​A-3′
	Reverse 5′-CTT​GGA​CCA​GCT​CTG​GAT​TTA​G-3′
*Irs1*	Forward 5′-GCC​AAT​CTT​CAT​CCA​GTT​GC-3′
	Reverse 5′-CAT​CGT​GAA​GAA​GGC​ATA​GG-3′
*Pik3r1*	Forward5′-CGAAAACACAGAAGACCAATACTCA-3′
	Reverse 5′-TCC​CTC​GCA​ATA​GGT​TCT​CG-3′
*Akt1*	Forward 5′-GCC​TCT​GCT​TTG​TCA​TGG​AG-3′
	Reverse 5′-AGC​ATG​AGG​TTC​TCC​AGC​TT-3′
*Foxo1*	Forward 5' - TCGAACCAGCTCAAACGC - 3'
	Reverse 5′-GGT​GGA​TAC​ACC​AGG​GAA​TG - 3'
*Sirt1*	Forward 5' - TAC​CAG​AAC​AGT​TTC​ATA​GAG​CCA​T - 3'
	Reverse 5' - CAA AAT​GTA​GAT​GAG​GCA​GAG​GTT- 3′
*G6pc*	Forward 5' - GCG​TGC​CAT​AGG​ACT​CAT​CA- 3'
	Reverse 5' - CAC​CAG​CAA​ACA​ATT​GCC​CA- 3'

### Statistical Analysis

**FIGURE 1:**
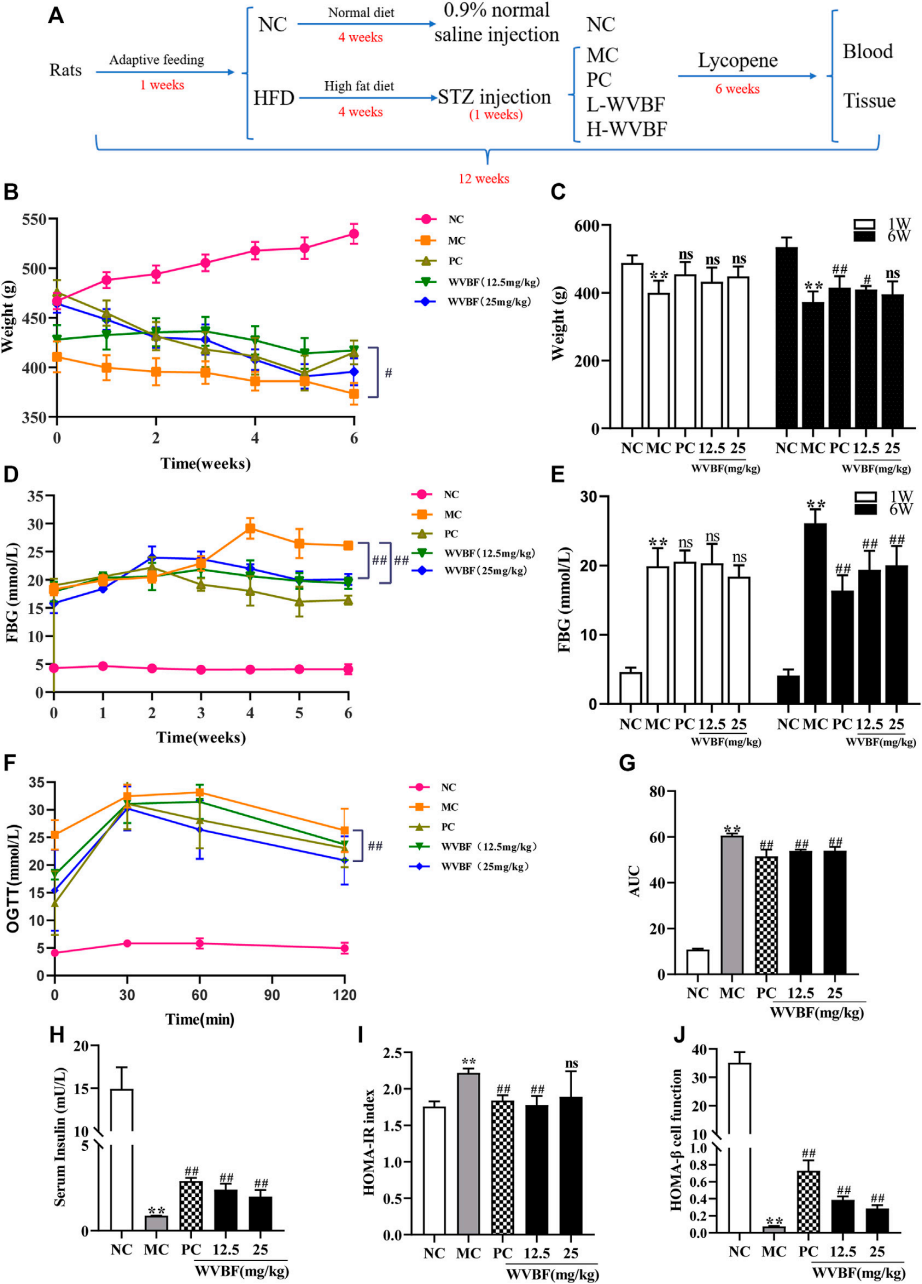
Effects of WVBF treatment on weight, blood glucose and insulin resistance of diabetic rats. **(A)** overview of the experimental process. **(B, C)** The effect of WVBF on the body weight of diabetic rats. **(D, E)** The level of FBG from one to 6 weeks **(D)** and at the end of 6 weeks **(E)**. **(F, G)** The levels of blood glucose **(F)** and areas under curves AUC **(G)** in oral glucose tolerance test (OGTT) at the end of 6 weeks **(H–J)** WVBF intervention influenced insulin sensitivity and insulin resistance in diabetic rats. HFD: high-fat diet, L-WVBF: WVBF 12.5 mg/kg, H-WVBF: WVBF 25 mg/kg, WVBF 12.5 mg/kg, NC: normal control, MC: mellitus control, PC: positive control, 12.5: WVBF 12.5 mg/kg/day, 25: 25 mg/kg/day. Date were shown as mean ± SEM; *p < 0.05, **p < 0.01 *vs*. NC group; #*p* < 0.05, ##*p* < 0.01, *vs*. MC group. B-E, *n* = 8 in each group; F-J, *n* = 6 in each group.

The data were expressed as mean ± standard error. Data were analyzed for statistical significance using Student’s t-test for unpaired observations. Statistical significance between the different groups was analyzed by a one-way ANOVA followed by least significant difference (LSD) test comparison post hoc test for normal distribution data. The significance level was fixed at *p* < 0.05. Data are presented as means ± SEM. *p < 0.05, **p < 0.01, two-tailed Student’s t-test (Figure 3A); one-way ANOVA followed by LSD post hoc test (Figure 1 and Figures 3C–I).

## Results

### WVBF Treatment Exerted Hypoglycemic Effect by Relieving Insulin Resistance in HFD and STZ-Treated Rats

To explore the effects of WVBF on liver injury and insulin resistance in diabetic rats, a desirable T2DM model that induced by HFD and STZ was proposed and its metabolic features were measured. As shown in Figures 1B,C, there was lower and slower body weight gain in MC than that in NC group rats, while WVBF or PC intervention significantly promoted the weight gain. As shown in Figures 1D,E, the blood glucose levels in three treatment groups, i.e. PC treatment group, WVBF 12.5 mg/kg and WVBF 25 mg/kg intervention groups were markedly decreased, compared with those in MC group at the end of 6-weeks treatment. The oral glucose tolerance test (OGTT) and area under the curve (AUC) demonstrated that WVBF treatment improved glucose tolerance compared with MC group at the end of 6 weeks (Figures 1F,G).

Next, we analyzed fasting insulin (FINS) index that reflects insulin resistance. The FINS level of MC group was significantly lower than that in NC group in the 6^th^ week (Figure 1H). WVBF (12.5 and 25 mg/kg) and PC treatment for 6 weeks increased FINS levels in rats. HOMA-IR and HOMA-β are two indexes used to evaluate insulin resistance and pancreatic *ß*-cell function. The HOMA-IR index was higher and the HOMA-β was lower in MC group than those in NC rats (Figures 1I,J). However, both WVBF (12.5 mg/kg, 25 mg/kg) and PC treatment alleviated insulin resistance and enhanced pancreatic *ß*-cell function, compared with the MC group. Together, these findings indicated that WVBF treatment ameliorated diabetes by increasing insulin secretion in HFD and STZ-induced rats.

### WVBF Treatment Improved Pathological Damage on Liver and Pancreas in Rats

**FIGURE 2:**
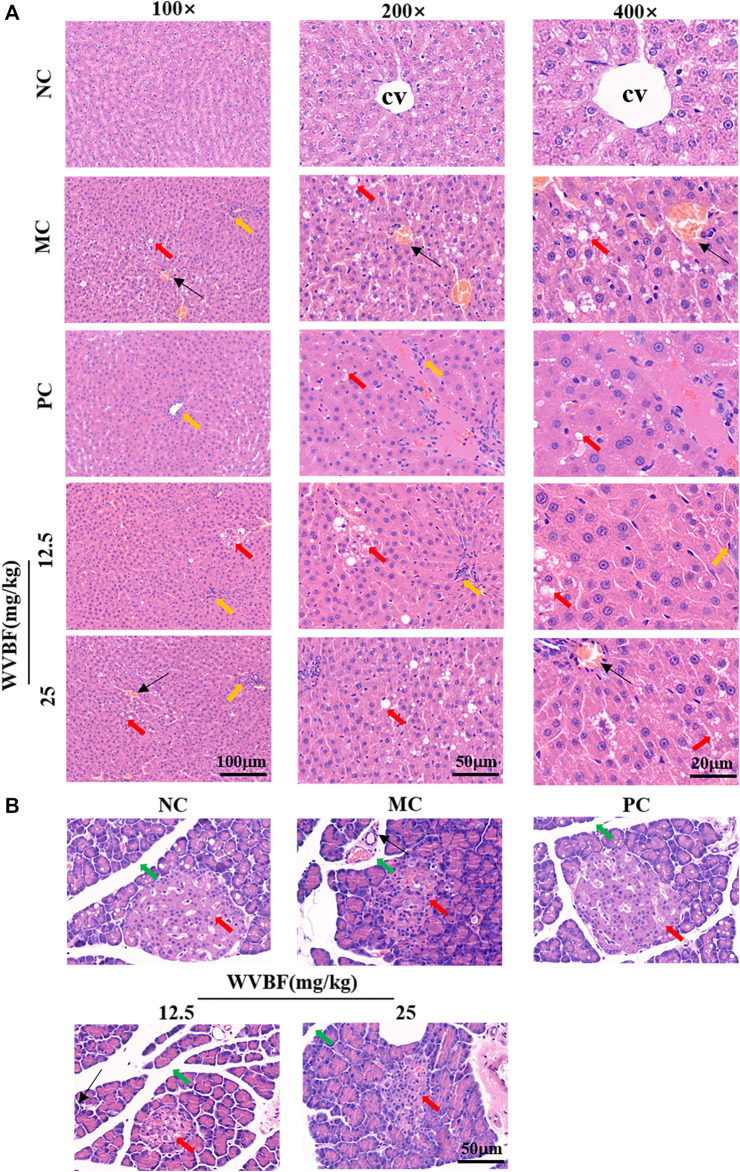
Effect of WVBF treatment on the hepatic and pancreatic morphology in diabetic rats **(A)**. H&E staining of liver from different groups. Upper panel, hematoxylin and eosin, 100×; middle panel, hematoxylin and eosin, 200×; Bottom panel, hematoxylin and eosin, 400×. CV indicates central vein; Red arrow indicates edema; Yellow arrow, inflammatory cells infiltration; Black arrow head indicates hyperemia. Bottom panel, **(B)**. H&E staining of pancreas from different groups. Red arrow, pancreatic acini; green arrow, intralobular duct of the pancreas; black arrow head, inflammatory cells. Hematoxylin and eosin, 100×. NC: normal control, MC: mellitus control, PC: positive control, 12.5: WVBF 12.5 mg/kg/day, 25: 25 mg/kg/day.

Herein, we further explored the effects of WVBF on liver and pancreas pathology in diabetic rats. H&E staining (Figure 2A) showed that elevated steatosis, edema, hyperemia, hepatocellular ballooning, and focal necrosis with inflammatory cells infiltration in MC group. The WVBF or PC treatment groups ameliorated pathological damage, compared with MC group. The pancreas is a main target organ with a key role in diabetic pathological conditions. As shown in Figure 2B, the islets were abnormally shaped, the borders were blurred, the acinar were broken, and the ducts were dilated and inflammatory cells infiltration in the MC group, these changes demonstrated that the islets were severely damaged. In WVBF treatment group, the islet shape was close to regular, the extent of dilation of lobule ducts and inflammatory infiltration were alleviated compared with MC group. Together, these results demonstrated the protective action of WVBF on diabetic organ injury of liver and pancreas.

### Transcriptome and Functional Assays Revealed WVBF Restores the Gene Expression of Insulin Resistance in Diabetic Rats

**FIGURE 3:**
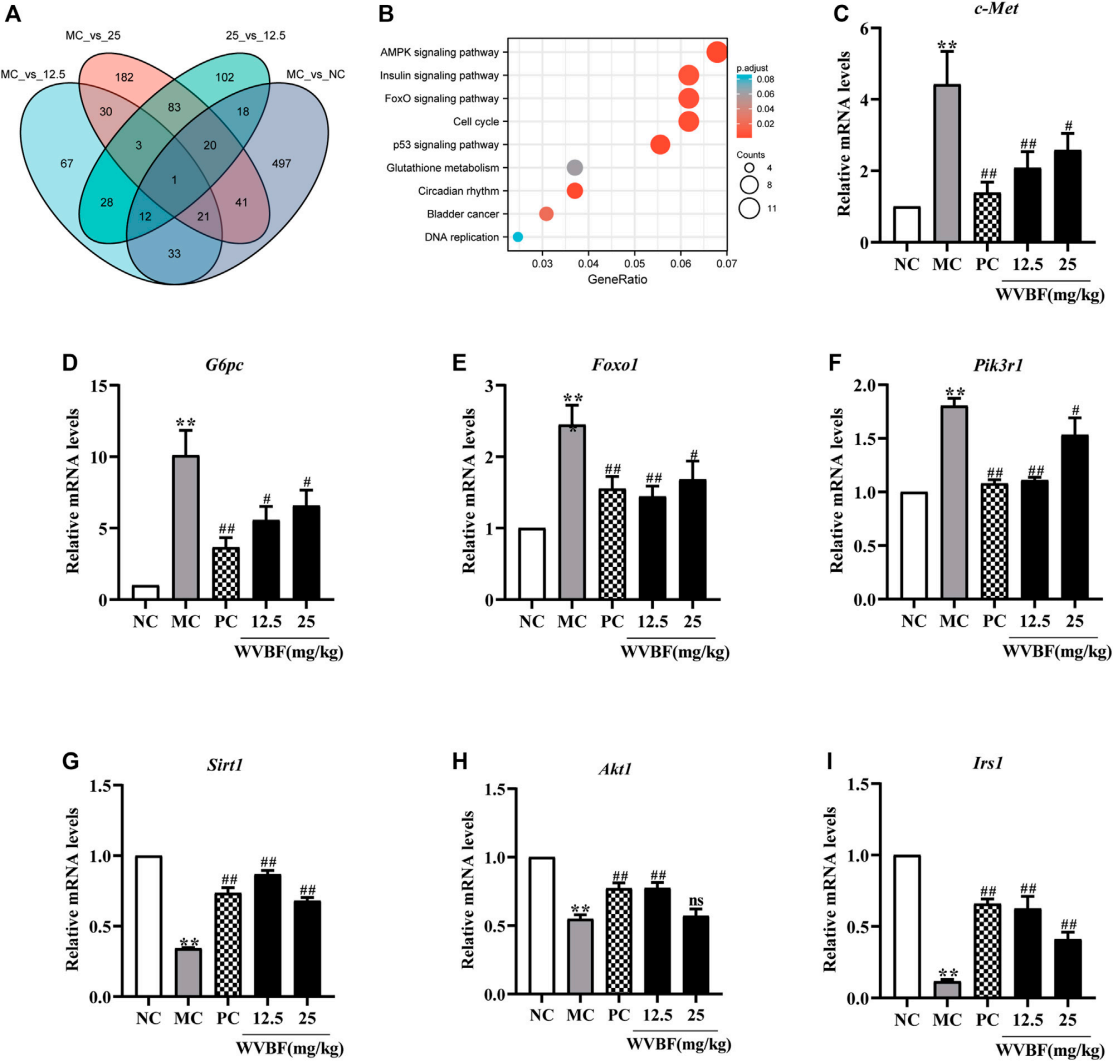
Screening and verification of differentially expressed genes in diabetic rats. **(A)** Venn diagram of differentially expressed genes (DEGs) in the different groups. **(B)** Enrichment analysis of Kyoto Encyclopedia of Genes and Genomes (KEGG) annotation signaling pathway (WVBF 25 mg/kg-treated versus MC). **(C–I)** The key gene (c-Met, G6pc, Foxo1, pik3r1, Sirt1, Akt1, Irs1) expression in liver tissues from different groups. NC: normal control, MC: mellitus control, PC: positive control, 12.5: WVBF 12.5 mg/kg/day, 25: 25 mg/kg/day. Date were shown as mean ± SEM; *p < 0.05, **p < 0.01 *vs*. NC group; #*p* < 0.05, ##*p* < 0.01, *vs*. MC group. *n* = 3 in each group.

To explore the molecular mechanisms of WVBF ameliorating diabetes, we analysed the transcriptome profiling of the liver in four groups. In liver, 13324 genes (fragments per kilobase of transcript per million fragments mapped [FPKM] > 1) were detected (Supplementary Table S1). We identified 643 differentially expressed genes (DEGs) in the comparison of MC groups versus NC groups, and 381 DEGs were detected in the WVBF (25 mg/kg)-treated versus MC groups (Figure 3A). The majority of the 162 genes with significant difference in expression were mainly related to insulin and inflammatory response (Insulin signaling pathway, FoxO signaling pathway, AMPK signaling pathway) (Figure 3B and Supplementary Table S2). The functional enrichment analysis indicated that significant differentially expressed genes (c-Met, Irs1, and G6pc) were enriched in these top three KEGG pathways. AMPK signaling pathway is associated with insulin resistance and metabolic syndrome-associated disorders (Xu et al., 2014). These findings revealed that WVBF treatment exerted anti-diabetic effects by inhibiting insulin resistance and improving the damage of liver and pancreas (Figure 2 and Figure 3B).

**FIGURE 4:**
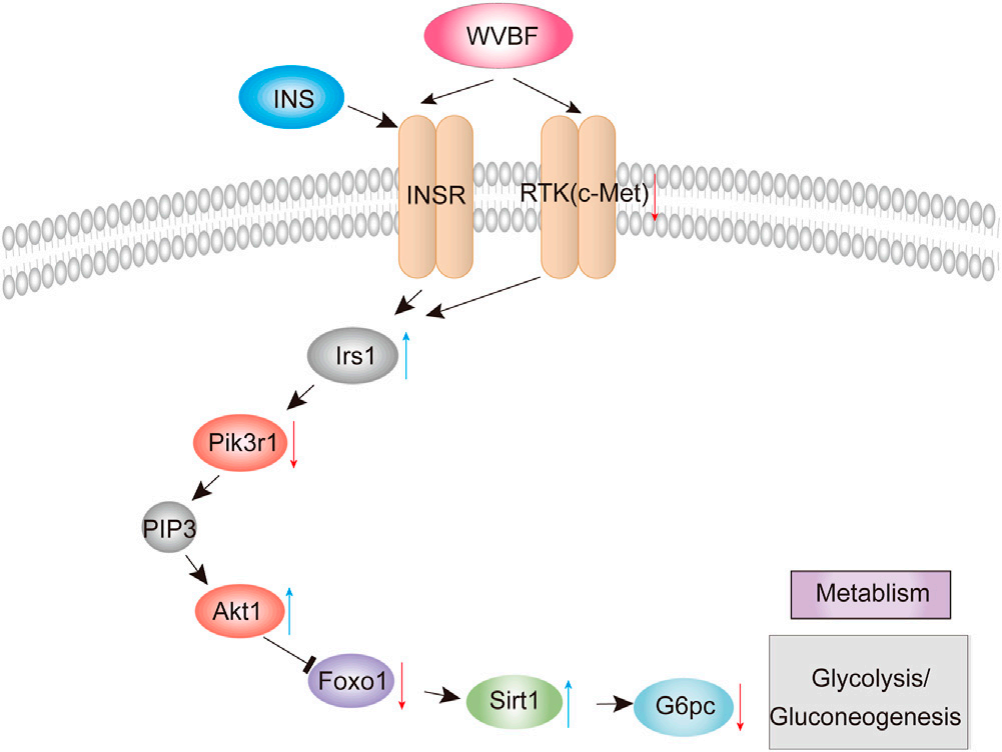
WVBF-mediated pathways in diabetic liver injury.

To validate the WVBF effects on ameliorating insulin resistance by aforementioned analysis, we also chose certain key molecules linking insulin signaling and glycolysis/gluconeogenesis, such as Pikr1, Akt1, Foxo1 and Sirt1 (Figure 4). Results of qRT-PCR indicated that WVBF treatment inhibited the levels of Foxo1, G6pc, c-Met and Pik3r1 in the liver, which were increased in the MC group (Figures 3C–I). As indicated, the expressions of genes related to metabolism and inflammation, including Sirt1, Irs1, Akt1, were significantly increased after WVBF treatment, which were down-regulated in the MC group (Figures 3G–I). Consistent with our RNA-seq results, WVBF treatment increased serum insulin levels and improved the damage of liver and pancreas (Figure 1 and Figure 2). IRS-1/PI3K/AKT pathway plays a central role in the metabolic actions of insulin to maintain glucose metabolic homeostasis (Taniguchi et al., 2006). Our previous work indicated that the connection of IRS-1/PI3K/AKT pathway in the beneficial actions of WVBF (He et al., 2020a). Therefore, these findings conformed that WVBF treatment ameliorated diabetic liver injury by reducing insulin resistance, which safeguarded the normal secretion of insulin to control blood glucose.

## Discussion

Accumulating studies demonstrate that many Chinese herbal medicines have beneficial effects on the treatment of metabolic disorders including diabetes (Seto et al., 2015). Herein, we established a type 2 diabetic rat model induced by a HFD-STZ. We further showed that the protective effect of WVBF on T2DM liver injury by RNA-sequencing and functional assays on the liver tissue of diabetic rats.

T2DM is a common metabolic disorders characterized by insulin resistance and pancreatic *ß* cell failure (Li J. et al., 2018). Consistently, elevating blood glucose levels may cause reduced peripheral insulin sensitivity and glucose intolerance (Lee et al., 2012). Insulin secreted by pancreatic *ß*-cells fails to meet the metabolic demand, leading to the development of T2DM (Kasuga, 2006). Insulin resistance is the main cause of liver injury, thereby ameliorating insulin resistance is an important way to improve the diabetic liver injury. Our previous studies have indicated that WVBF intervention enhanced effectively insulin sensitization along with protection of liver, anti-oxidation and improved glucose metabolism in mice (He et al., 2020a). Furthermore, *V. baillonii* exert insulin-mimicking effects by inducing phosphorylation of Akt and inhibit Pck1 mRNA levels in hepatoma cells (Huang et al., 2016). Moreover, WVBF ameliorates lipid accumulation by mediating oxidative, inflammatory and lipid metabolic signaling pathways in LO2 cells (Huang et al., 2020). In this present study, by integrating transcriptome and functional assays, we reveals that WVBF exert the anti-diabetic effect to improve diabetic organ injury of liver and pancreas in rats.

Our previous work, two doses of WVBF (25 and 50 mg/kg) were administered orally in different groups of mice in the presence or absence of WVBF and the protective effect of WVBF on diabetic liver injury were evaluated (He et al., 2020b). Moreover, our another study has shown that high doses of 100 mg/kg and 50 mg/kg WVBF were not always dose-dependent, with a certain inhibitory effect (Yu et al., 2016). Therefore, two doses of 12.5 and 25 mg/kg were used in this study to explore the protective effect of WVBF in diabetic rats. Our findings showed that the protective effect of 12.5 mg/kg WVBF was significantly better than 25 mg/kg WVBF in relieving insulin resistance and improving pathological damage on liver and pancreas. Improved glycaemic control is generally accompanied by weight gain (Ness-Abramof and Apovian, 2005; Liu et al., 2012). Elvert *et al.* reported that alterations in body weight during antihyperglycaemic treatment are closely related to the mechanism of action by which the drug controls blood glucose (Elvert et al., 2013). Pancreatic *ß*-cells express the gluconeogenic enzymes glucose 6-phosphatase (G6Pase), which modulate glucose-stimulated insulin secretion through their ability to reverse otherwise irreversible glycolytic steps (Westermeier et al., 2019). In this present study, WVBF intervention significantly improved body weight and insulin secretion in diabetic rats.

To validate the WVBF effects on ameliorating diabetic liver injury, this study demonstrated that WVBF treatment alleviated liver and pancreas islet injury by mediating the insulin signaling cascade (insulin and FoxO signaling pathway), which orchestrated the normal secretion of insulin to control blood glucose (Figure 4). c-Met is a key modulator that effectively manipulate insulin resistance in diabetes (Oliveira et al., 2018). The pancreas c-Met deficiency accelerates the onset of diabetes in multiple low-dose streptozotocin-injected mice (Mellado-Gil et al., 2011). c-Met deficiency in pancreas is associated with defective insulin secretion (Fagerholm et al., 2014). Insulin bind to the insulin receptor, subsequently activate insulin receptor substrate (IRS) proteins, initiating the classical insulin signaling pathway (Du and Wei, 2014). IRS1 belongs to the IRS protein family and is responsible for transferring the insulin/IGF1-signaling. In liver, IRS1 is important to mediate insulin dependent regulation of glucose and lipid metabolism and complement in the diurnal regulation thereof (Eckstein et al., 2017). IRS1 also integrates the feedback signals and communicates with other signaling pathways, which triggers the onset of insulin resistance (Thirone et al., 2006). In this present study, WVBF treatment increases gene expressions of Irs1 significantly, which alleviate insulin resistance in diabetic rats.

PI3K-Akt signaling is the various mechanistic links in metabolic disorders, including diabetes (Krycer et al., 2010). PIK3R1, an important candidate gene, exhibits a vital role in insulin signal transduction of T2DM progression (Karadogan et al., 2018). Slience of Pik3r1 surppresses insulin resistance in diet-induced obesity (DIO) mice (McCurdy et al., 2012). Akt1, a serine/threonine kinase, was found to improve glucose metabolism, when activated in models of DIO and age-related fat accumulation (Cheng et al., 2015). IRS-1/PI3K/AKT/FOXO1 pathway plays a central role in the metabolic actions of insulin to maintain glucose metabolic homeostasis (Taniguchi et al., 2006). Foxo1 is a key regulator that mediated by insulin under physiological conditions in type 2 diabetic mice (Ge et al., 2021). An insulin-independent mechanism for transcriptional regulation of Foxo1 in type 2 diabetic mice. Furthermore, Battiprolu *et al.* found that FoxO1-dependent decrease of IRS1 induced the inactivation of Akt signaling and alleviation of insulin resistance (Battiprolu et al., 2012). Foxo1-SIRT1 signaling pathway plays key roles in insulin resistance induced by T2DM (Sin et al., 2015). SIRT1 has been implicated in obesity, insulin resistance, type 2 diabetes mellitus and fatty liver disease (Zhou et al., 2018). Sirt1 upregulation may serve as a potent therapeutic approach against development and progression of diabetic complications (Strycharz et al., 2018). Various signaling pathways governed by insulin converge at the level of transcriptional regulation of the key hepatic gluconeogenic genes G6PC, this as one of the focal mechanisms through which gluconeogenesis is modulated (Hatting et al., 2018). Moreover, WVBF inhibited the higher G6Pase mRNA levels in diabetic rats, suggesting that WVBF alleviating insulin resistance in part by inhibiting gluconeogenesis in diabetic rats. G6pc, a key enzyme in the glucose metabolism expressed primarily in the liver. Insulin can regulate hepatic gluconeogenesis via transcription of genes involved in gluconeogenic control, including G6PC (Resaz et al., 2011). Interestingly, our findings indicated that WVBF treatment exert a protective role in diabetes by affecting insulin signaling cascade. Therefore, this study implicated that WVBF could manipulate insulin homeostasis by orchestrating both insulin production and insulin utilization.

In conclusion, this present study uncovered that WVBF could alleviate HFD-STZ -induced diabetic liver injury *in vivo*, and improve insulin resistance in rats. WVBF treatment exhibited the protective effects on liver injury in diabetic rats, which restored the gene expression of insulin resistance and insulin signaling cascade. These findings provide a valuable therapy option for the well-known WVBF, which could be used as a promising therapeutic agent in treating diabetes and its complications.

## Data Availability

The datasets presented in this study can be found in online repositories. The names of the repository/repositories and accession number(s) can be found below: Raw sequence data reported in this study have been deposited in the publicly accessible Genome Sequence Archive of the BIG Data Center (https://bigd.big.ac.cn/gsa) , Beijing Institute of Genomics (BIG), Chinese Academy of Sciences, under the accession number, PRJCA006659.
